# The making of a branching annelid: an analysis of complete mitochondrial genome and ribosomal data of *Ramisyllis multicaudata*

**DOI:** 10.1038/srep12072

**Published:** 2015-07-17

**Authors:** M. Teresa Aguado, Christopher J. Glasby, Paul C. Schroeder, Anne Weigert, Christoph Bleidorn

**Affiliations:** 1 Departamento de Biología, Facultad de Ciencias, Universidad Autónoma de Madrid, Cantoblanco, 28049 Madrid, Spain; 2 Museum and Art Gallery of the Northern Territory, GPO Box 4646, Darwin, N.T., Australia; 3 School of Biological Sciences, Washington State University, Pullman, Washington 99163-4236, USA; 4 Molecular Evolution and Systematics of Animals, Institute of Biology, University of Leipzig, Talstraße 33, D-04103 Leipzig, Germany; 5 Max Planck Institute for Evolutionary Anthropology, Deutscher Platz 6, 04103 Leipzig, Germany

## Abstract

*Ramisyllis multicaudata* is a member of Syllidae (Annelida, Errantia, Phyllodocida) with a remarkable branching body plan. Using a next-generation sequencing approach, the complete mitochondrial genomes of *R. multicaudata* and *Trypanobia* sp. are sequenced and analysed, representing the first ones from Syllidae. The gene order in these two syllids does not follow the order proposed as the putative ground pattern in Errantia. The phylogenetic relationships of *R. multicaudata* are discerned using a phylogenetic approach with the nuclear *18S* and the mitochondrial *16S* and *cox1* genes. *Ramisyllis multicaudata* is the sister group of a clade containing *Trypanobia* species. Both genera, *Ramisyllis* and *Trypanobia*, together with *Parahaplosyllis*, *Trypanosyllis, Eurysyllis,* and *Xenosyllis* are located in a long branched clade. The long branches are explained by an accelerated mutational rate in the *18S* rRNA gene. Using a phylogenetic backbone, we propose a scenario in which the postembryonic addition of segments that occurs in most syllids, their huge diversity of reproductive modes, and their ability to regenerate lost parts, in combination, have provided an evolutionary basis to develop a new branching body pattern as realised in *Ramisyllis*.

Annelids are a taxon of marine lophotrochozoans with mainly segmented members showing a huge diversity of body plans^1^. One of the most speciose taxa is the Syllidae, which are further well-known for their diverse reproductive modes. There are two reproductive modes in Syllidae, called epigamy and schizogamy, and both modes involve strong anatomical and behavioural changes. Epigamy is considered the plesiomorphic reproductive mode. It results in significant morphological and behavioural changes in the benthic, sexually mature adults^2^, which undergo enlargement of anterior appendages and eyes and development of swimming notochaetae in midbody-posterior segments. Notochaetae are absent in non-reproductive syllids, which only bear neurochaetae for locomotion. Transformed individuals actively ascend to the pelagic realm where spawning occurs. After spawning, the animals usually die, though some are able to reverse these changes and go back to the benthic realm for further reproductive activity. Schizogamy, the putatively derived mode, produces reproductive individuals or stolons from newly produced posterior segments. The stolons develop eyes and their own anterior appendages, as well as special swimming (noto-) chaetae, while they maintain attached to the parental body. When they are completely mature, they are detached from the parental stock for swimming and spawning^2^,^3^,^4^,^5^. Finally, the stolons die after spawning. Meanwhile, the stock remains in the benthic realm, thereby avoiding the dangers of swimming into the pelagic zone, and will be able to reproduce more than once. Schizogamy can be subdivided into scissiparity (only one stolon is developed in a reproductive cycle), or gemmiparity (several stolons are developed simultaneously during the same reproductive cycle)^3^,^4^,^5^. Some gemmiparous syllids, like *Myrianida* Milne Edwards, 1845 (a member of Autolytinae), are able to produce a series of stolons, one after another (Fig. 1A), with the last one being the most developed and the first to be detached^6^. Another type of gemmiparity has been described for some species of Syllinae: *Trypanosyllis* Claparède, 1864, *Trypanobia* Imajima & Hartmann, 1964 (Recently erected from subgenus to genus level), and *Parahaplosyllis* Hartmann-Schröder, 1990^7^,^8^,^9^,^10^,^11^,^12^,^13^,^14^,^15^,^16^,^17^. These animals are able to produce numerous stolons in collateral or successive budding, i.e. growing dorsally or laterodorsally from the last segment or from several posterior most segments (Fig. 1B–D). In addition, *Trypanosyllis* and *Trypanobia* have been reported to live in a symbiotic association with other animals, like echinoderms and sponges. Among them, the genus *Trypanobia* is the best known symbiotic form, with species such as *T. asterobia* (Okada, 1933) living within the body of the sea star *Luidia quinaria* von Martens, 1865 and developing the stolons within the host^12^.

Remarkably, within Syllinae, two species have been described with a morphology that makes them unique among all so far ~20,000 described annelids: *Ramisyllis multicaudata* Glasby, Schroeder and Aguado, 2012 and *Syllis ramosa* McIntosh, 1879. These animals are the only two branching annelids^18^,^19^,^20^ and they live in strict symbiosis within sponges (Fig. 2A,B). They have one “head” but multiple branches (Fig. 2C,D); each of them goes into a canal of their host, growing within the sponge by producing new branches and enlarging the existing ones. This body pattern and biology has astonished biologists and the general public since they were first described. The feeding behaviour providing nutrition for these large syllids is unknown and it remains unclear if they prey on their host. *Ramisyllis multicaudata* and *S. ramosa* also reproduce by stolons that are developed at the end of terminal branches (Fig. 1E,F). Initial phylogenetic analyses found that *R. multicaudata* is related to the genera *Parahaplosyllis*, *Trypanosyllis*, *Eurysyllis* Ehlers, 1864, and *Xenosyllis* Marion & Bobretzky, 1875^18^. These genera, together with *Trypanobia*, show a dorsoventrally flattened or ribbon-shaped body. The analysis performed by Glasby *et al.* (2012)^18^ only included the sequences of 16S and a fragment of the 18S, which was difficult to amplify for *R. multicaudata* using standard primers. However, the phylogenetic placement of this branching annelid is a prerequisite to understand the evolution and character transformations leading to this unique body plan.

Mitochondrial genomes are valuable sources of phylogenetically informative markers. The mitochondrial genome is, in most animals, a circular duplex molecule of DNA, approximately 13–17 kb in length, with 13 protein coding genes (*nad1-6, nad4L, cox1-3, cob, atp6/8*), 22 tRNAs (*trnX*), two rRNAs (*rnS, rnL*), and one AT-rich non-coding region, the control region (CR), which is related with the origin of replication^21^. The order of these genes is subject to several types of rearrangements, such as inversion, transposition, and tandem duplication random loss, which is a duplication of several continuous genes, followed by random loss of one copy of each of the redundant genes^21^. The high number of combinations found in Metazoa suggests that the order of genes is relatively unconstrained^22^.

Mitochondrial gene order has been used for phylogenetic inference in several animal taxa at different taxonomic levels^23^,^24^. Because of high substitution rates of nucleotides and amino acids between taxa, and biases in nucleotide frequencies, the usefulness of mitochondrial sequences for deep phylogenies seems to be restricted^25^. However, they are well suited to recover phylogenetic relationships of younger divergences^25^. Complete mitochondrial genomes of annelids are only available for a limited number of taxa and, as far as we know, all genes are transcribed from the same strand^26^. The gene order is considered as generally conserved in this taxon and most investigated annelids differ only in a few rearrangements of tRNAs, which are usually regarded as more variable^21^. To date, around 40 complete mitochondrial genomes have been published, representing three of the main lineages (Errantia, Sedentaria, Sipuncula) within the group^27^. No mitochondrial genomes have been published so far for the taxon Syllidae (Errantia).

The advent of next generation sequencing techniques has simplified the generation of mitochondrial genomes, which now can be assembled as a by-product from whole genome sequencing. Due to the high copy number of mitochondria per cell, even low coverage genome data enabled the reconstruction of complete organelle genomes^28^. Hence we used short read Illumina sequencing for the generation of whole genome shotgun (wgs) data for two syllid species. Based on genome assemblies we extracted the mitochondrial and, additionally, nuclear ribosomal data, for phylogenetic analysis. Based on this data, we resolve the phylogenetic relationships of *R. multicaudata* using a molecular phylogenetic approach and extensive taxon sampling. As initial analyses suggested a possible close relationship with the genus *Trypanobia*, we also generated wgs data for this taxon. Moreover, the complete mitochondrial genomes of *R. multicaudata* and *Trypanobia* sp. are compared and the derived phylogeny is used to develop an evolutionary scenario leading to a branching annelid.

## Results

### Phylogenetic analyses

The first noticeable result to be indicated is that the *18S* sequence obtained herein from the sequencing library of *Ramisyllis multicaudata* does not match with the one amplified by PCR and sequenced in 2012 (Genbank Acc. n° JQ292795)^18^. The *18S* from our genome assembly is considerably longer than those of many other syllids (>2200 bp) and shows large insertions in the V2- and V5 region. However the sequences from *16S* obtained herein from the mt genome and the one already published (Genbank JQ313812) were identical. Analysing carefully the small piece of *18S* sequence JQ292795 and also the sequence of its proposed sister group, *Parahaplosyllis brevicirra* Hartmann-Schröder, 1990 (Genbank JF903679), we found suspicious similarities with the *18S* sequence of another syllid, *Syllis alternata* Moore, 1908 (Genbank JF903649). We consider these as possible contaminations since they were sequenced at the same time. Hence, the sequence from *18S* obtained herein was used to replace the previous one (JQ292795), which together with *P. brevicirra* (JF903679) were excluded from the analyses of the *18S* partitions.

The ML analysis of the first, most inclusive data set (large *18S*, Fig. 3), shows *R. multicaudata* as sister to *Trypanobia* sp. and *Trypanobia depressa* (Augener, 1913) (100% bootstrap), and related to other genera, such as *Trypanosyllis*, *Xenosyllis* and *Eurysyllis*, the same relatives as proposed by Glasby *et al.* (2012)^18^. All these taxa are in a maximally supported clade (100% bootstrap) that shows a conspicuously long branch. In order to dismiss possible Long Branch Attraction effects (LBA) and make the alignments easier, a second group of data sets was analysed focussing on a smaller taxon sampling (the trimmed *18S*, *16S* and *cox1*, respectively). The trees for each independent analysis show congruent topologies (Supplementary Figure 1A–C). *Ramisyllis multicaudata, Trypanobia* sp. and *T. depressa* are closely related and nested in a larger clade together with *Trypanosyllis*, *Eurysyllis*, *Xenosyllis,* and *Parahaplosyllis* (*16S* and *cox1* trimmed partitions, Supplementary Figure 1B,C). The *18S* topology (Supplementary Figure 4A) reveals again a long branch for this clade, while in *16S* and *cox1* it is not longer than others (Supplementary Figure 1B,C). In *16S* and *cox1* partitions, *P. brevicirra* is close to its previously proposed relatives^18^. The combined data set (trimmed *18S* + *16S* + *cox1*) recovers *R. multicaudata* closely related to *Trypanobia* sp. and *T. depressa* (100% bootstrap) (Fig. 4A). The *Ramisyllis-Trypanobia* clade is sister to a clade containing *Eurysyllis tuberculata* Ehlers, 1864 and *Xenosyllis scabroides* San Martín, Aguado & Hutchings, 2008. *Parahaplosyllis brevicirra* is sister to *Trypanosyllis* sp. and *Trypanosyllis zebra* (Grube, 1860) (94% bootstrap). *Trypanosyllis coeliaca* Claparède, 1868 is not located within this latter group. In all analyses, the genus *Trypanosyllis* is paraphyletic.

### Mitochondrial and nuclear genomes

The assembly of the *R. multicaudata* whole genome shotgun data resulted in 278,762 contigs with an N50 of 769 bp and an average GC content of 32%. The assembly of *Trypanobia* sp. resulted in 40,225 contigs with an N50 of 676 bp and an average GC content of 38%. As the coverage turned out to be too low for analysing *k*-mer abundances, an approximate genome size estimation was inferred from estimating the coverage of single copy ribosomal protein genes. Based on the average coverage of these genes we estimate a size ~500 mbp for *Trypanobia* sp. and a genome size of ~1500 mbp for *R. multicaudata*. BLAST-searches for putative contigs of the sponge host genome revealed no signs of possible contamination from host tissue, as for example due to feeding.

BLAST-searches identified the complete mitochondrial genomes of both investigated syllids as a single contig, which in both cases represented the longest contig of the low coverage genome assembly. The complete mitochondrial genome of *R. multicaudata* is 15,748 bp long and is assembled with a coverage of 105x; the one of *Trypanobia* sp. is 16,630 bp long and has a coverage of 70x. The two genomes are AT-rich (67% in *R. multicaudata*, 69% in *Trypanobia* sp.); A is the most common base (34% in *R. multicaudata*, 36% in *Trypanobia* sp.), and G the least common (12% and 11%, respectively). In both genomes, the coding strand has a strong skew of G vs. C (−0.31 in *R. multicaudata*, −0.29 in *Trypanobia* sp.), whereas the AT skew is positive (0.02, 0.04, respectively). Both mt genomes contain the same 37 genes as found in most other annelids and typically present in bilaterian mt genomes: 13 protein-coding genes, two genes for rRNAs, and 22 genes for tRNAs (Supplementary Table 2, 3). As in the case for all annelids so far studied, all genes are transcribed from the same strand (referred to as plus-strand). However, huge differences were found in the gene arrangement of *R. multicaudata* and *Trypanobia* sp. when compared with the other known annelids^26^ (Fig. 5) and the putative ground pattern of Bilateria^25^. Additionally, none of the conserved blocks for Bilateria proposed by Bernt *et al.* (2013)^25^ have been found in the syllids.

The CREx analyses (Supplementary Figure 2,3) showed that the differences between *Lumbricus terrestris* Linnaeus, 1758 (representing Sedentaria) and *Platynereis dumerilii* (Audouin & Milne Edwards, 1834) (representing Errantia), each with *R. multicaudata* could be explained by 5 tandem duplication random losses (tdrl), respectively. Differences between *L. terrestris* and *P. dumerilii*, each with *Trypanobia* sp. could be explained by 4 tdrl and 1 transposition, respectively. Finally, differences between *R. multicaudata* and *Trypanobia* sp. could be explained by 1 transposition and 4 tdrl; and differences between *L. terrestris* and *P. dumerilii* by 1 transposition and 3 reversals. In addition, the matrix of a gene order similarity measure (Supplementary Table 4) reveals that the gene order between *R. multicaudata* and *Trypanobia* sp. is more different than the order of *L. terrestris* and *P. dumerilii.* Considering the order of only protein coding genes, *R. multicaudata* and *Trypanobia* sp. differ in one transposition (Fig. 5). In summary, the two mitochondrial genomes of the closely related syllids here investigated show more differences between themselves than exist between *L. terrestris* and *P. dumerilii*, the latter which might be separated by some hundred million years.

The putative control region in *R. multicaudata* is 702 bp in length and flanked by *nad6* and *trnL1* (Supplementary Table 1, Fig. 5). In *Trypanobia* sp., the control region is 782 bp and it is located between *trnS2* and *trnL1* (Supplementary Table 2, Fig. 5). In *R. multicaudata*, besides the control region, 27 other non-coding regions are dispersed over the whole genome, ranging from one to 147 base pairs; the largest one located between *trnG* and *rrnL* (Supplementary Table 1). In *Trypanobia* sp., there are 34 non-coding regions in addition to the putative control one, ranging from 1 to 265 base pairs, the largest one between *trnY* and *trnM* (Supplementary Table 2).

Start codons in protein-coding genes are highly biased towards ATG (Supplementary Table 3). In *R. multicaudata*, ATG is observed in 10 of 13 coding genes; other start codons are ATA and ATT. In *Trypanobia* sp., ATG is observed in 11 of the 13 coding genes; other start codons are ATT and TTG. In *R. multicaudata*, the dominant stop codon is TAA, except 1 gene ending in TAG, and one putatively incomplete stop codon ending in only T (Supplementary Table 1). In *Trypanobia* sp., the most used stop codon is also TAA, except 1 gene were it is TAG (Supplementary Table 2). There is also a codon usage bias in both genomes (Supplementary Table 3). In general, NNG codons are the least used, while especially NNA, followed by NNT codons are the most common codon types.

The typical 22 tRNAs were found in both mt genomes. They mostly possess the common cloverleaf structure, with an acceptor arm, anticodon arm, TΨC arm, DHU arm, and associated loop regions (Supplementary Figure 4,5). In *R. multicaudata*, the DHV stem is missing in *trnC* and *trnR*; in *Trypanobia* sp. the DHV stem is missing in *trnR*. *Ramisyllis multicaudata* and *Trypanobia* sp. have *trnS1* and *trnS2* with a shortened DHV stem. The sizes of the ribosomal RNAs are, in *R. multicaudata*, *rrnL*: 1008 bp, *rrnS*: 787 bp; and in *Trypanobia* sp., *rrnL*: 1007 bp, *rrnS*: 789 bp. The two genes are not separated by any tRNA, only by an intergenic spacer of 20 and 27 bp in *R. multicaudata* and *Trypanobia* sp., respectively (Supplementary Table 1, 2).

## Discussion

Our phylogenetic analyses find *Ramisyllis multicaudata* and *Trypanobia* sp. within Syllidae closely related to other genera (*Parahaplosyllis*, *Trypanosyllis*, *Xenosyllis* and *Eurysyllis*) in a long branched clade (Figs 3 and 4, Supplementary Figure 1). The genera *Parahaplosyllis*, *Trypanosyllis*, *Eurysyllis*, *Xenosyllis*, and *Trypanobia* share a distinct dorsoventrally flattened body, referred to as ribbon-like shaped^29^. This feature might represent a synapomorphy of this clade, though there is a reversal in *R. multicaudata*, which exhibits a cylindrical body pattern. This characteristic is used here to name the long branched clade including *R. multicaudata* as the “ribbon” clade (Fig. 4A).

The phylogenetic results shown herein are broadly congruent with a preliminary analysis^18^, even though the *18S* sequence of *R. multicaudata* in that publication seems to be a contamination from another syllid species. Highly covered ribosomal sequences from the whole genome shotgun approach showed derived *18S* sequences, when compared with other syllids. As such, the *18S* of *R. multicaudata* is considerably longer, containing several long insertions. These differences may explain the failure when trying repeatedly to sequence *18S* from *R. multicaudata* in 2012. Analyses of single gene partitions show that the *18S* gene in particular greatly contributes to the observed long branches of the ribbon clade. Analyses solely based on the mitochondrial genes reveal congruent topologies, but with considerably shorter branch lengths. Interestingly, a previous study mentioned a big effort when sequencing *18S* from *R. multicaudata*, with sequences from the host sponge recovered repeatedly^18^. However, BLAST searches against high copy number genes of the sponge (e.g., ribosomal genes, mitochondrial genes) did not identify any sponge related contigs in our whole genome assembly. We therefore suggest that *R. multicaudata* is not feeding on its host, even though the way these worms support their large body mass with nutrition remains unclear.

The analysed wgs data do not only reveal highly derived ribosomal sequences, leading to long branches in our analyses. Similarly, the mitochondrial gene order found in *R. multicaudata* and *Trypanobia* sp. also differs considerably in comparison with the gene order of Sedentaria (*L. terrestris*) and Errantia (*P. dumerilii*) (Supplementary Figure 2,3). Annelids of both these major groups analysed so far showed a remarkably conserved gene order, suggesting a common ground pattern (Fig. 5). Indeed, the differences between the gene order of Errantia, Sedentaria, the basal branching annelid lineages (represented by sipunculids), and the putative ground pattern of Spiralia (Bernt *et al.*, 2013) are less drastic than those between any of these patterns and the analysed syllids (Fig. 5, Supplementary Table 4). In the light of these results, we conclude that the gene order in the mt genome of (Pleisto-) Annelida is more diverse than expected. Our results indicate that there might be some constraints that maintain the gene order, reflected in the relatively conserved patterns of annelid lineages; but once these constraints are violated, many changes seem possible, as revealed by the patterns in *R. multicaudata* and *Trypanobia* sp. However, since these are the first two mitochondrial genomes from syllids, it is not possible to assess if Syllidae in general, or only members of the ribbon clade including *Ramisyllis* and *Trypanobia*, show these huge differences.

Other mitochondrial genome features of *R*. *multicaudata* and *Trypanobia* sp. seem to be more in line with those of other annelids. Both species share with other annelids a similar pattern of codon usage bias (NNA and NNT the most common types, while NNG the least used)^30^. A negative GC-skew is also found in most of the mitochondrial genomes known from annelids. Regarding the tRNAs, DHU stems are lacking in many metazoan mitochondrial tRNA genes^22^. The sizes of the ribosomal RNAs in *R. multicaudata* and *Trypanobia* sp. are within the size range of other invertebrates including molluscs and annelids. The two genes are not separated though usually, in many animals, *trnV* is found in the middle; among annelids only echiurans (*Urechis caupo* Fisher & MacGinitie, 1928) and myzostomids (*Myzostoma seymourcollegiorum* Rouse & Grygier, 2005) share this condition^31^.

Low coverage, whole genome analyses of the two syllids allowed several interesting findings. Both taxa not only share an unusual biology, but also show derived ribosomal sequences and a strongly changed mitochondrial gene order. Rough estimations of the nuclear genome size from mapping sequence reads on putatively single copy ribosomal proteins indicates a genome size between 500 mbp and 1.5 gbp for both these taxa. In the case of *Ramisyllis* this size seems to be considerably larger than that of most other syllid annelids, which ranges between 100 and 500 mbp^32^. In summary, these results suggest a labile genomic architecture of the ancestral lineages of these taxa, putatively driven by genetic drift which could point to several past population size bottlenecks^33^.

The branching body pattern of *Ramisyllis* might be the result of the combination of processes that are closely related: post-embryonic development and regeneration. In many groups of marine annelids, segment formation occurs during both larval and juvenile development and is the result of the activity of a *posterior growth zone* located immediately anterior to the pygidium^34^. The *posterior growth zone* has been also referred to as *segment addition zone* (SAZ)^35^, and this latter term will be followed herein. Usually, in marine annelids with an unlimited number of segments bodies grow indeterminately, adding segments throughout their whole life^35^. The SAZ may contain teloblasts, which are progenitor cells that divide to produce new segments^36^. In these dividing cells, the cleavage plane must be perpendicular to the main axis (antero-posterior axis or A-P axis), to proliferate according to bilateral symmetry. In addition to the continuous segment formation, many groups within annelids are able to regenerate lost segments. Posterior regeneration processes imply the generation of a new SAZ^36^. On the molecular level, gene expression during adult growth and regeneration shows several similarities, suggesting a shared mechanism^37^,^38^. Interestingly, the occasional occurrence of annelids with two tails have been recorded both in nature, and, more frequently, in worms in which regeneration has been studied experimentally^39^,^40^,^41^,^42^. These aberrant forms might be the result of punctual “mistakes” or induced experiments during the regeneration process; and there are no reports of these animals living further for reproductive activity. Considering the occurrence of these aberrant branching forms, branching in *Ramisyllis* might be the result of a similar unknown event, which could have been established as a regular growth pattern.

*Ramisyllis* is a member of Syllidae, which might suggest that an additional process could be involved. Syllids are animals with a high capacity of regeneration of lost parts; they can regenerate anteriorly as well as posteriorly, and they show a derived mode of reproduction called schizogamy that involves this regeneration ability^5^,^14^. Schizogamy implies the ability to produce reproductive individuals or stolons from newly produced segments, and also the ability to regenerate the posterior end after detachment of stolons^5^. The regeneration of the parental stock begins with a pygidium and a new SAZ. Therefore, the life cycle in schizogamous syllids is a combination of segment addition during adult life, reproduction and regeneration.

The recovered phylogeny shown herein (Fig. 4A,B) can be used to develop a scenario of major character transformations leading to a branched annelid species. The herein analysed *R. multicaudata* and *Trypanobia* sp. are both members of Syllinae, characterized, among other features, by a schizogamous reproductive mode. In addition they are both members of the ribbon clade, where species reproduce by gemmiparity; i.e. the development of simultaneous stolons during the same reproductive cycle^3^,^4^,^5^ (Fig. 4A). There is one report of *Parahaplosyllis* as gemmiparous^16^, one report of *Trypanobia*, and several of *Trypanosyllis* species^7^,^8^,^9^,^10^,^11^,^12^,^13^,^14^,^15^,^16^,^17^. Other species in these genera reproduce by scissiparity or their reproductive mode has not been identified yet. Additionally, it is unknown if gemmiparity and scissiparity are exclusive of each species or if a species can alternate between the two modes.

**Figure 1:**
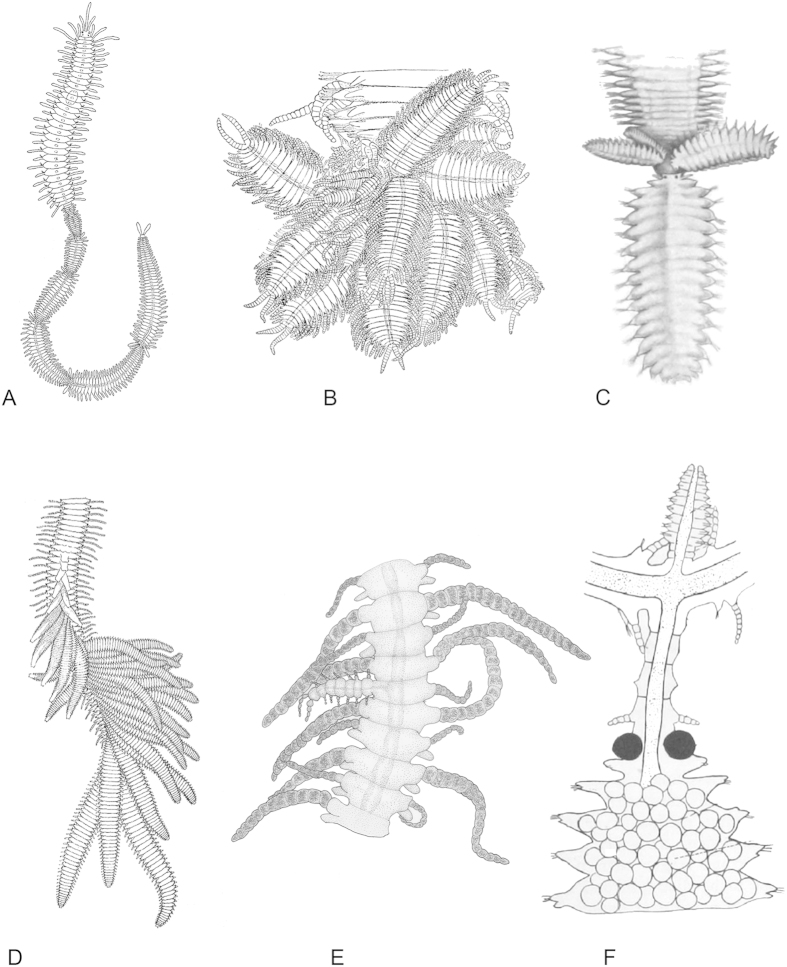
Different modes of gemmiparity in Syllidae and budding in *Syllis ramosa*. **A**. Sequential gemmiparity in *Myrianida* sp. (modified after Okada, 1933^12^); **B**. Colateral budding in *Trypanosyllis gemmipara* Johnson, 1901 (modified after Johnson, 1902^8^); **C**. Collateral budding in *Trypanosyllis crosslandi* Potts, 1911 (modified after Potts, 1911^9^); **D**. Successive budding in *Trypanobia asterobia* (modified after Okada, 1933^12^); **E**. Development of a lateral branch in *S. ramosa* (drawing by MT Aguado from the holotype); **F**. Branches and stolon in *S. ramosa* (modified after McIntosh, 1885^20^). All figures used for modifications are part of the public domain.

In gemmiparous species of *Trypanosyllis* and *Parahaplosyllis* the stolons are developed from the parental body through the same posterior SAZ (Fig. 1B,C). However, stolons in *T. asterobia* are produced by separate consecutive parental segments (Fig. 1D). This evidence implies that *Trypanobia* develops successive SAZ simultaneously. In both, collateral budding (in *Trypanosyllis* and *Parahaplosyllis*) and successive budding (*Trypanobia*), the newly produced segments are developed from each SAZ at different angles from the A-P axis, being most evident in *Trypanobia* (Fig. 1D). The stolon attachment to the parental stock dorsally or dorsolaterally from the stock might suggest that the cleavage plane of division in the proliferating cells is somehow rotated; however, this hypothetical process is completely unknown to date.

**Figure 2:**
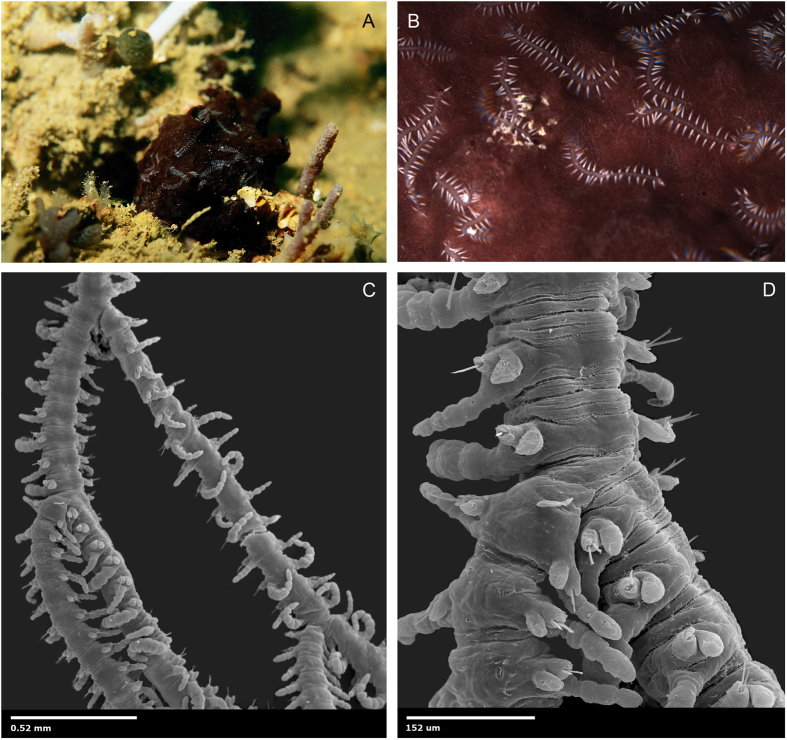
**A**. Living sponge, *Petrosia* sp. with posterior ends of *Ramisyllis multicaudata* emerging from surface pores and moving actively on the sponge surface; **B**. Detail of *R. multicaudata* posterior ends on the surface of the sponge; **C**. *R. multicaudata*, non-type, SEM of branch points, mid-body region; **D**. *R. multicaudata*, non-type, SEM, detail of one midbody branch point. All photographs were taken by CJ Glasby and PC Schroeder by SEM with methods as described previously^18^.

**Figure 3:**
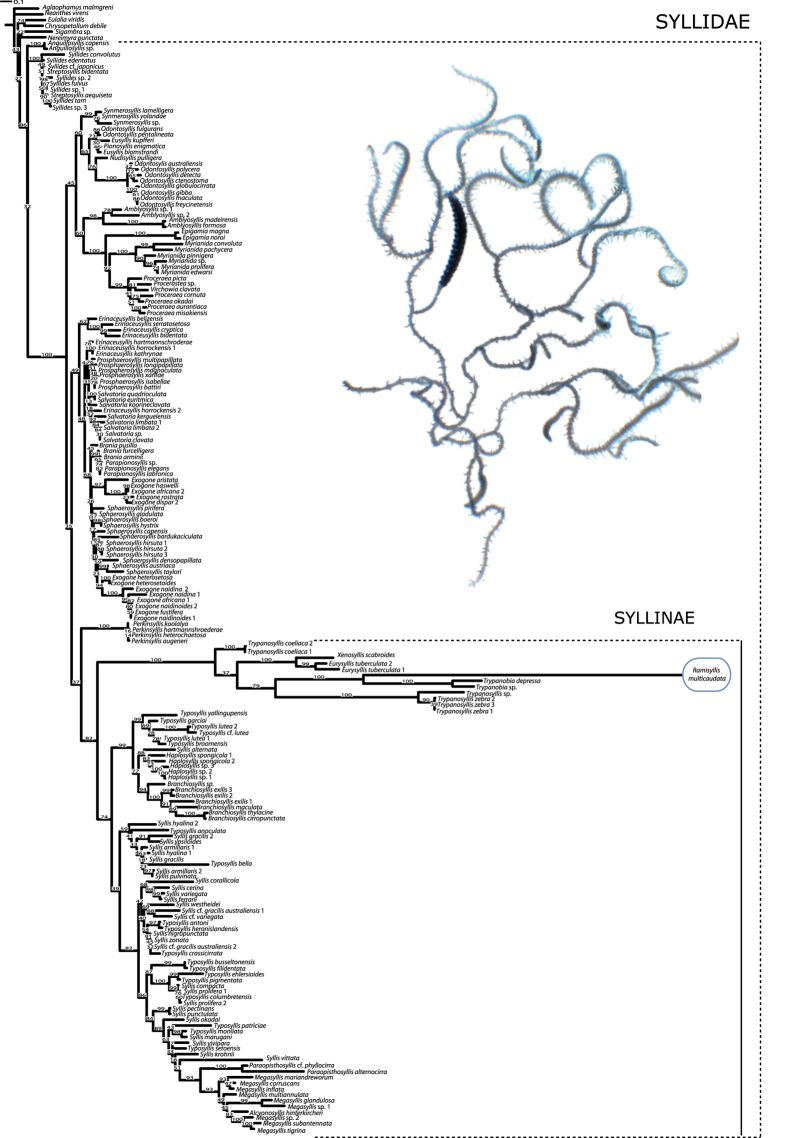
Maximum likelihood tree obtained when analysing the complete *18S* partition. Bootstrap support values are above nodes. Picture of *Ramisyllis multicaudata* with stolons taken by CJ Glasby.

**Figure 4:**
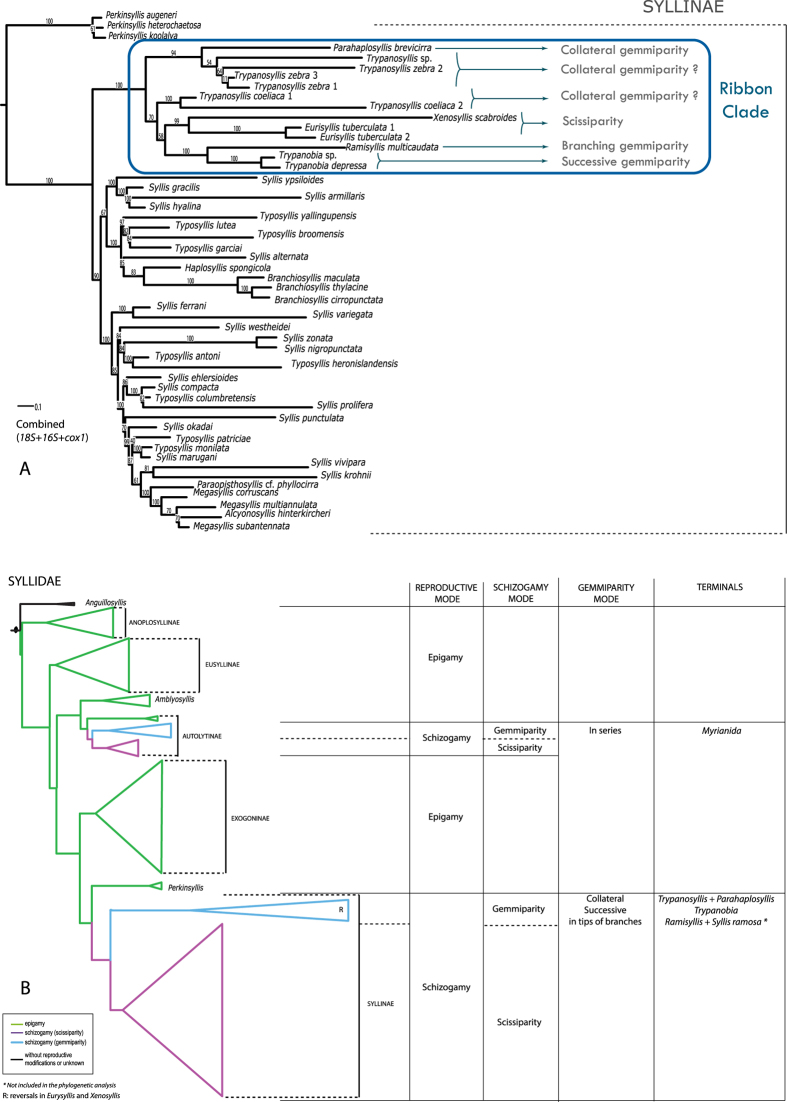
**A**. ML tree obtained when analysing the combined trimmed data set (*18S* + *16S* + *cox1*). Reproductive modes in the ribbon clade are shown. The reproductive mode found in some species is assumed for the genera they belong. Collateral budding is dubious in *Trypanosyllis* since the gemmiparous species might be related to any or both groups (*T. zebra*-*Trypanosyllis* sp. and *T. coeliaca*). **B**. Reproductive modes in Syllidae. Phylogenetic relationships based on the analysis of the gene *18S* (Fig. 2). Reproductive modes are assumed at generic level.

**Figure 5:**
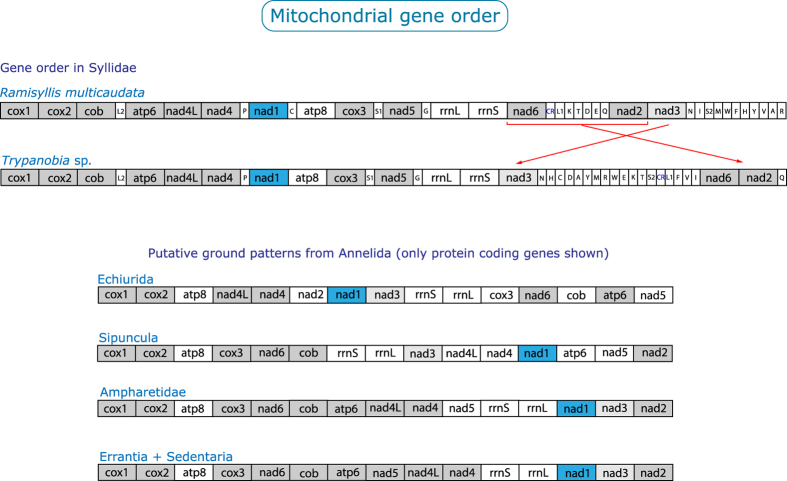
Mitochondrial gene order in *Ramisyllis multicaudata* and *Trypanobia* sp. Putative ground patterns for Annelida after Golombek *et al.* (2013)^26^.

A similar process might be responsible for development of the body pattern in *R. multicaudata*, since the asymmetrical branches in its body suggest different active SAZs; however, time for proliferation might be longer and instead of producing the stolons directly from the newly produced segments, these arise only at the tip of terminal branches. Additionally, in *Ramisyllis* the development of SAZs is random, while in *Trypanobia* they are developed in successive posterior segments. In *Ramisyllis*, branches occur asymmetrically and laterally from the A-P main axis. The reproductive mode of *R. multicaudata* and *S. ramosa* could be also considered a particular type of gemmiparity, since they are able to produce several stolons simultaneously; at the tip of terminal branches of its body^18^,^19^,^20^ (Figs 1F and 2A,B).

Summarizing all these observations, and assuming that the reproductive modes found in some species is representative for the genera they belong, a possible scenario might be proposed in which an ancestor of the ribbon clade reproduced by collateral gemmiparity (as seen in species of *Parahaplosyllis* and *Trypanosyllis*) (Fig. 1B,C). This condition could have been later modified in the ancestor of *Trypanobia* and *Ramisyllis*, which might also maintain symbiotic relationships with other organisms (Fig. 2A,B). This hypothetical ancestor might have been able to develop several simultaneous SAZs, each producing an asymmetrical branch of new segments. The newly developed segments are directly transformed into stolons in *Trypanobia* (Fig. 1D), or more segments are added in a growing large chain in *R. multicaudata* (Fig. 1E,F), and only the distal ones are transformed into stolons; the latter strategy appears to be an adaptation to life in the complex canal system of a sponge. Within the ribbon clade, gemmiparity reverses into scissiparity in *Eurysyllis* and *Xenosyllis* (Fig. 1A,B).

An alternative hypothesis may explain the branching pattern as an independent process from the schizogamous reproductive mode, and hence, considering the phylogenetic results, an autopomorphy of *Ramisyllis*. Future comparative studies on the genetic basis of the stolonization processes and branching body patterns of these species could give further support to any of these scenarios.

## Conclusions

In this study, we provide the first complete mitochondrial genomes for Syllidae and present a scenario for the evolution of the unique morphology of the branching syllid *Ramisyllis multicaudata*. We find that *R. multicaudata* and *Trypanobia* are sister taxa closely related to *Parahaplosyllis, Trypanosyllis, Eurysyllis, and Xenosyllis* and herein called the ribbon clade. The two closely related investigated species reveal strongly rearranged mitochondrial gene orders, unique for annelids. Both a comparatively large nuclear genome and strongly rearranged mitochondrial genomes could indicate past population size bottlenecks as such labile genomic architectures are more likely to be driven by genetic drift. We recognize that major phenotypic changes in syllid annelids are known from experimental studies on regeneration. The combination of undetermined postembryonic addition of segments, high ability to regenerate lost parts and gemmiparity might represent the basis for the development of a branching body pattern.

Macroevolutionary questions require complex answers integrating processes from multiple scales, ranging from within genomes to among species^43^. The available evidence obtained in this study suggests that *R. multicaudata* represents an example of major phenotypic transitions occurring by saltatational evolution^44^,^45^. Future analyses unravelling the genetic basis for the development of this unique body plan will provide further evidence for or against this hypothesis and hence we will be able to discern if *Ramisyllis* is indeed a *hopeful monster.*

## Materials and methods

### Genome sequencing and analyses

Sample collection from Darwin Harbour (Australia) and preparation of *Ramisyllis multicaudata* was previously described^18^. Scanning Electron Microscope (SEM) images were taken with a Hitachi S-570 microscope. *Trypanobia* sp. was collected at Lizard Island (northern Great Barrier Reef, Queensland, Australia) during a Polychaete Workshop in 2013. DNA was extracted from a single individual of each *R. multicaudata* and *Trypanobia* sp. by proteinase K digestion and subsequent chloroform extraction. For Illumina sequencing, double index sequencing libraries with average insert sizes of around 300 bp were prepared as previously described^46^. The libraries were sequenced as 125 bp paired-end run for *R. multicaudata* and 96 bp paired-end run for *Trypanobia* sp., both on an Illumina Hi-Seq 2000. Base calling was performed with freeIbis^47^, adaptor and primer sequences were removed using leeHom^48^, and reads with low complexity and false paired indices were discarded. Raw data of all libraries were trimmed by removing all reads that included more than 5 bases with a quality score below 15. The quality of all sequences was checked using FastQC (http://www.bioinformatics.babraham.ac.uk/projects/fastqc/, last accessed February 17th, 2015). *De novo* genome assemblies were conducted with IDBA-UD 1.1.046^49^, using an initial *k*-mer size of 21, an iteration size of 10 and a maximum *k*-mer size of 81. N50 and average GC-content of genome assemblies were evaluated using QUAST^50^. All sequence data were submitted to the National Centre for Biotechnology Information (NCBI) sequence read archive under accession numbers SRR2006110 for *R. multicaudata* and SRR2006109 for *Trypanobia* sp. Assembled and annotated mitochondrial genomes can be found on NCBI Genbank under accession numbers KR534502 for *R. multicaudata* and KR534503 for *Trypanobia* sp. Approximate genome size was estimated by mapping reads of single copy ribosomal proteins and subsequent coverage estimation using the software segemehl under default options^51^. The coverage of mitochondrial genomes was estimated by mapping sequence reads back to the contig comprising the mitochondrial genome using the same software. Possible contaminations of the *R. multicaudata* assembly due to its sponge host species (*Petrosia* sp.) were searched for using blastn searches. Three high copy number genes (*18S*, *28S*, *cox1*) published for *Petrosia* (*Strongylophora*) *strongylata* Thiele, 1903 (KF576656, KC869619) and *Petrosia ficiformis* (Poiret, 1789) (JX999088) served as queries.

### Phylogenetic analyses

The nuclear *18S* rRNA gene was retrieved from assemblies of *R. multicaudata* and *Trypanobia* sp. and was used in the phylogenetic analyses. Other terminals (Supplementary Table 1) were mostly included in previous analyses^2^,^52^,^53^. Two more species, *Trypanobia depressa* and *Trypanosyllis* sp. were included for the first time in these analyses; both were also collected in Lizard Island. The genes *18S*, *16S* and *cox1* from these two species were obtained using DNA extraction, primers, and amplification, and Sanger based sequencing procedures as specified previously^2^,^54^. Alignments were performed using the program MAFFT^55^ with the iterative refinement method E-INS-i, and default gap open and extension values. Several data sets were examined: 1. One set of *18S* sequences from a large number of syllids (195 terminals, Supplementary Table 5); 2. Trimmed sets of the partitions *18S*, *16S* and *cox1* with a reduced taxon sampling (51, 48 and 21, respectively) were used in order to simplify the alignments and dismiss possible Long Branch Attraction effects (LBA); 3. A combined data set of the trimmed partitions (*18S* + *16S* + *cox1*) (52 terminals). Each partition (large *18S* and trimmed *18S*, *16S* and *cox1* genes) was analysed independently, as was the combined molecular trimmed data set (*18S* + *16S* + *cox1*). Concatenation of partitions for the combined data set was conducted with FASconCAT^56^. Maximum Likelihood (ML) analyses were conducted using RAxML version 8.1.2^57^ with the GTR + I + G model. Bootstrap support values were generated with a rapid bootstrapping algorithm for 1000 replicates in RAxML.

### Mitochondrial genome annotation and analyses

AT and GC skews were determined for the complete mitochondrial genomes (plus strand) according to the formula AT skew = (A−T)/(A + T) and GC skew = (G−C)/(G + C), where the letters stand for the absolute number of the corresponding nucleotides in the sequences^58^. Characterization of codon usage bias was calculated with the program DAMBE5^59^.

The mitochondrial (mt) genomes of *R. multicaudata* and *Trypanobia* sp. were annotated using the MITOS webserver^60^ with the invertebrate mitochondrial code (NCBI code). This server also provided the secondary structure of tRNAs and rRNAs. To compare mitochondrial gene orders, we included the complete mt genomes of the earthworm *Lumbricus terrestris* and the nereidid polychaete *Platynereis dumerilii*, downloaded from GenBank with the accession numbers NC_001673 and AF178678, respectively. These two species were chosen as representatives of the Sedentaria and Errantia respectively, the two lineages comprising most of the species diversity of Annelida^27^. The mt genomes of *L. terrestris* and *P. dumerilii* were also annotated using the MITOS webserver^60^ with the invertebrate mitochondrial code (NCBI code). Finally, all automatic annotations were manually edited. We used CREx^61^ to conduct pairwise comparisons of the mitochondrial gene order of *R. multicaudata* and *Trypanobia* sp. with *L. terrestris* and *P. dumerilii*, respectively. CREx determines the most parsimonious genome rearrangement scenario between the gene order of each pair of taxa including transpositions, reverse transpositions, reversals, and tandem-duplication-random-loss (tdrl) events.

### Note added in proof

*Trypanobia* (previously a subgenus of *Trypanosyllis*) has been recently erected to genus level^62^.

## Additional Information

**How to cite this article**: Aguado, M. T. *et al.* The making of a branching annelid: an analysis of complete mitochondrial genome and ribosomal data of *Ramisyllis multicaudata*. *Sci. Rep.* **5**, 12072; doi: 10.1038/srep12072 (2015).

## Supplementary Material

Supplementary Information
