# Supplementary material for: The making of a branching annelid: an analysis of complete mitochondrial genome and ribosomal data of Ramisyllis multicaudata
Source: Sci Rep. 2015 Jul 17;5:12072. doi: 10.1038/srep12072 (PMC4505326; doi:10.1038/srep12072)
Supplement: Supplementary Information [file srep12072-s1.pdf]

Supplementary Information

The making of a branching annelid: an analysis of complete mitochondrial genome and ribosomal data of *Ramisyllis multicaudata*

M. Teresa Aguado[1*], Christopher J. Glasby[2], Paul C. Schroeder[3], Anne Weigert[4,5] and Christoph Bleidorn[4*]

[1] Departamento de Biología, Facultad de Ciencias, Universidad Autónoma de Madrid, Cantoblanco, 28049 Madrid, Spain

[2] Museum and Art Gallery of the Northern Territory, GPO Box 4646, Darwin, N.T., Australia

[3] School of Biological Sciences, Washington State University, Pullman, Washington 99163-4236, USA

[4] Molecular Evolution and Systematics of Animals, Institute of Biology, University of Leipzig, Talstraße 33, D-04103 Leipzig, Germany

[5] Max Planck Institute for Evolutionary Anthropology, Deutscher Platz 6, 04103 Leipzig, Germany

* corresponding: bleidorn@uni-leipzig.de, maite.aguado@uam.es

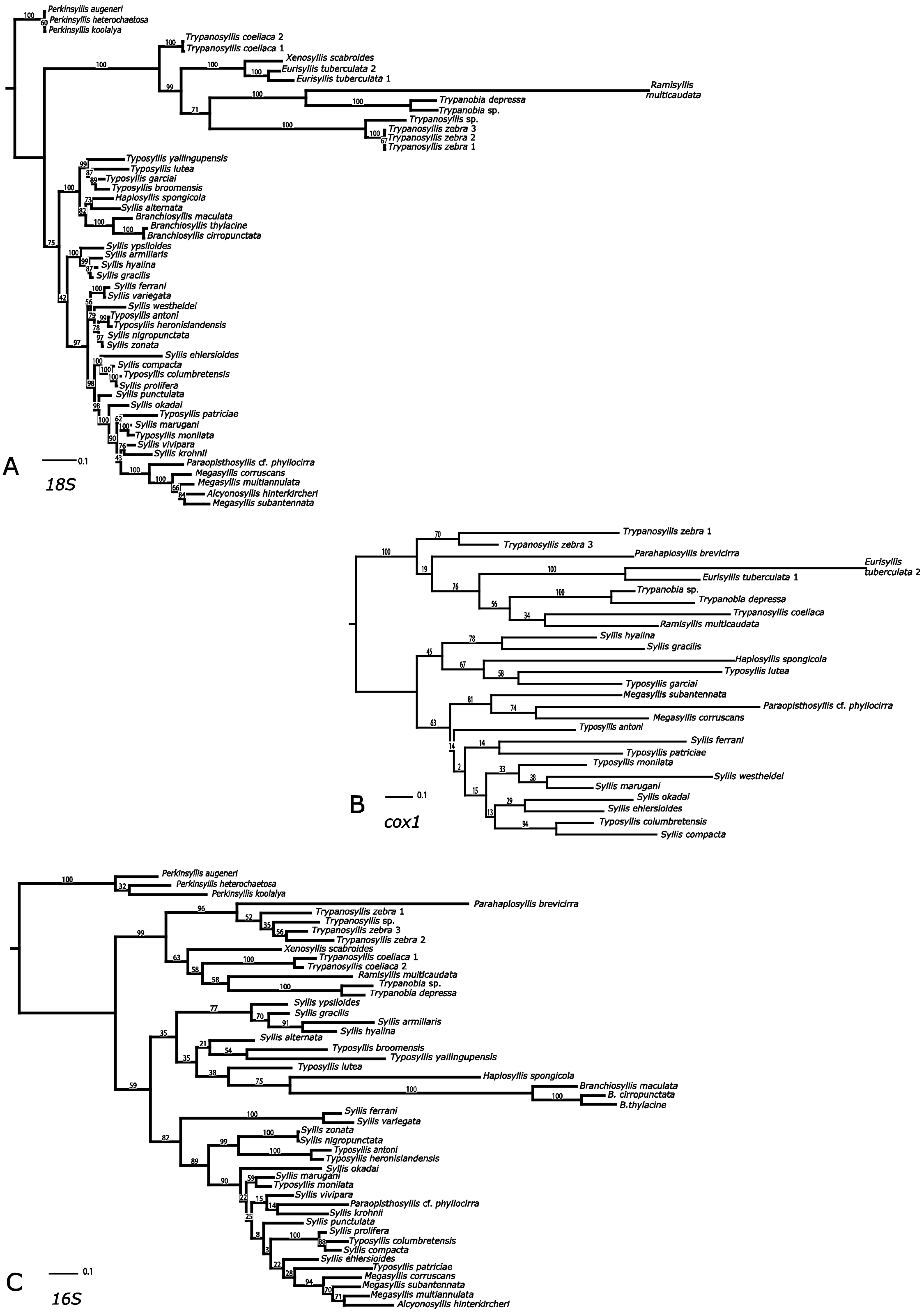

Supplementary Figure 1. Maximum likelihood trees from trimmed data sets, bootstrap support values are above nodes. A. ML tree obtained when analysing the *18S* trimmed partition; B. ML tree obtained when analysing the *cox1* trimmed partition. C. ML tree obtained when analysing the *16S* trimmed partition.

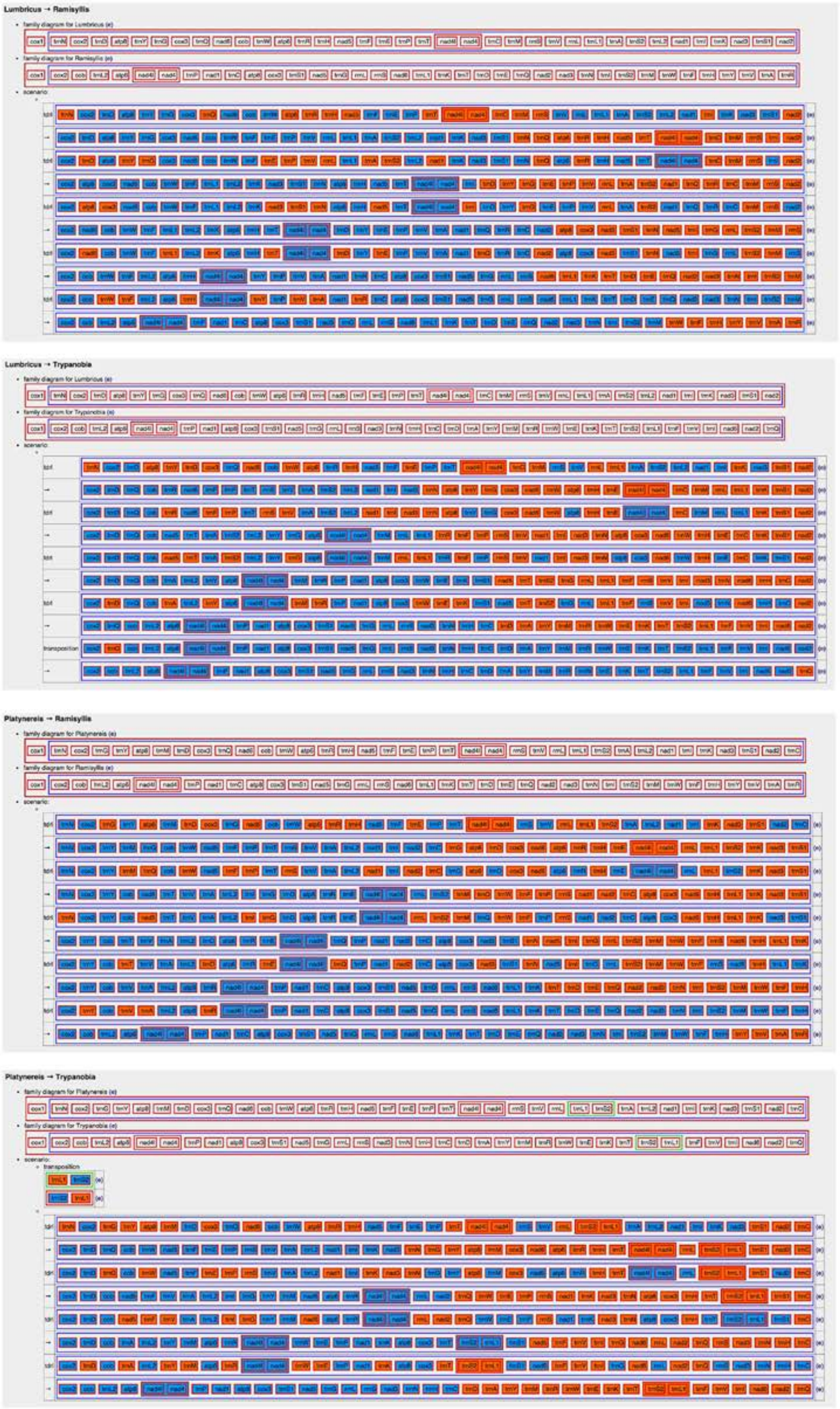

Supplementary Figure 2. A. Gene order comparison between *Lumbricus terrestris* and *Ramisyllis multicaudata*; B. Gene order comparison between *L. terrestris* and *Trypanobia* sp.; C. Gene order comparison between *Platynereis dumerilii* and *R. multicaudata*; D. Gene order comparison between *P. dumerilii* and *Trypanobia* sp.

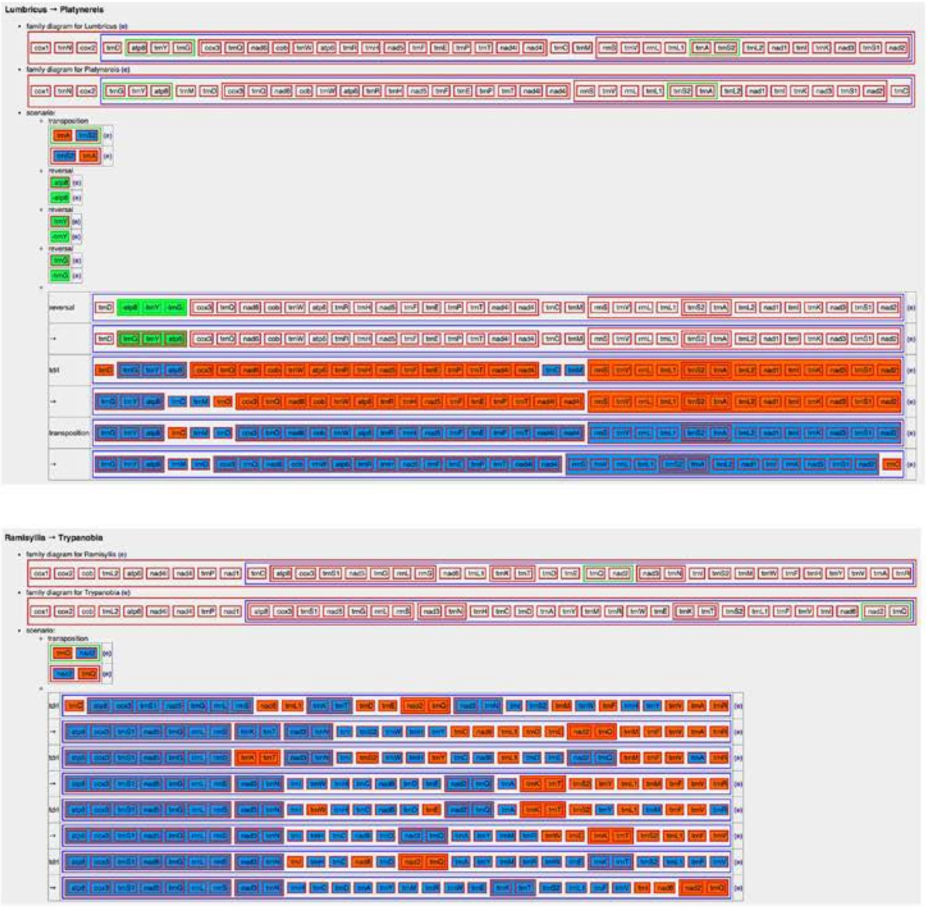

Supplementary Figure 3. A. Gene order comparison between *Lumbricus terrestris* and *Platynereis dumerilii*; B. Gene order comparison between *Ramisyllis multicaudata* and *Trypanobia* sp.

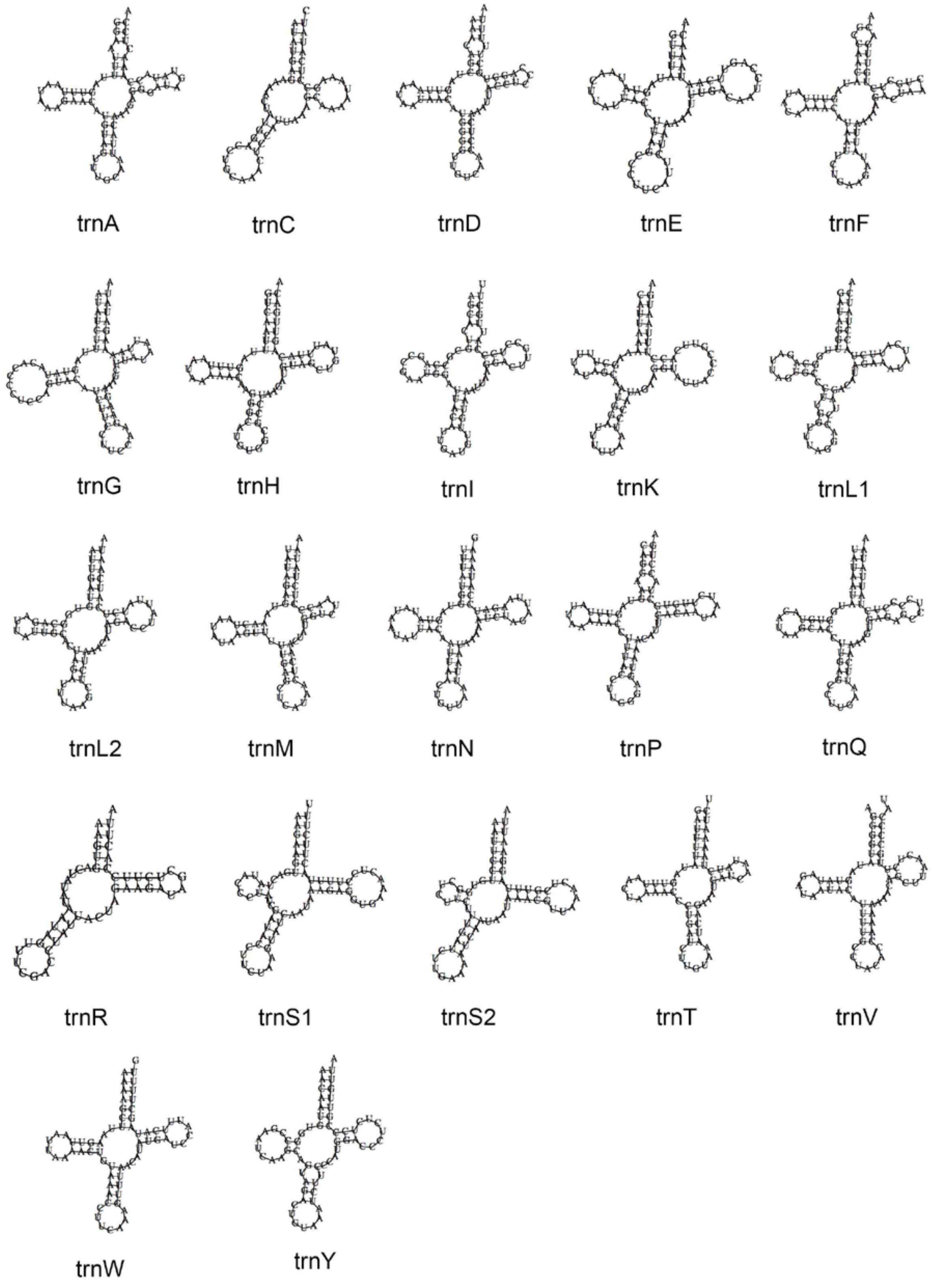

Supplementary Figure 4. Secondary structure of tRNAs of *Ramisyllis multicaudata*.

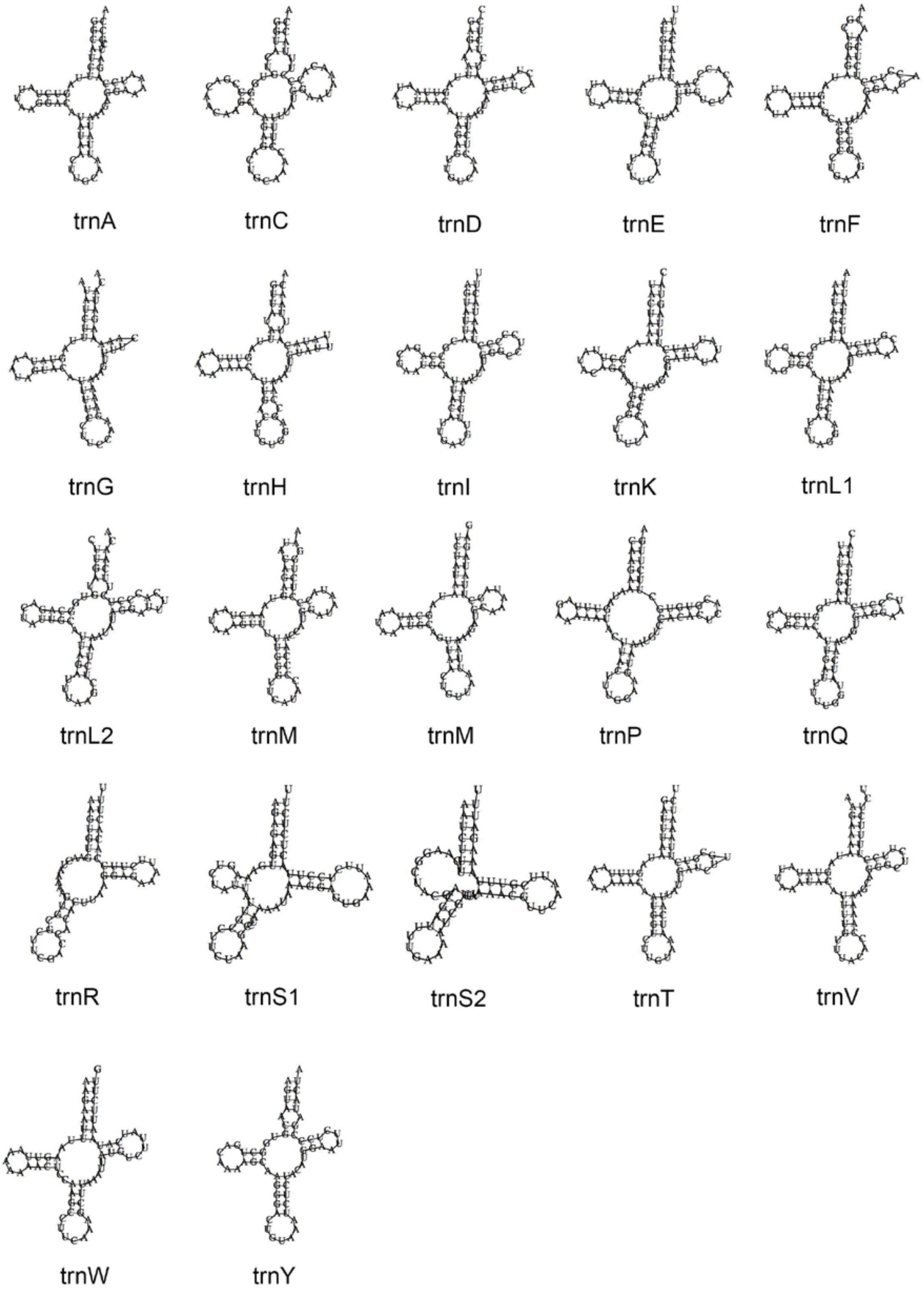

Supplementary Figure 5. Secondary structure of tRNAs of *Trypanobia* sp.

Supplementary Table 1. Gene content of the mitochondrial genome of *Ramisyllis multicaudata*

Gene	Start position	Stop position	Strand	Size (bp)	Start Codon	Stop codon
cox1	1	1536	+	1536	ATG	TAA
cox2	1537	2235	+	699	ATG	TAA
Intergenic spacer 1	2236	2240	+	5	n.a.	n.a.
cob	2241	3377	+	1136	ATG	TA
trnL2(taa)	3376	3439	+	64	n.a.	n.a.
Intergenic spacer 2	3440	3446	+	7	n.a.	n.a.
atp6	3447	4133	+	687	ATG	TAA
nad4l	4125	4429	+	305	ATT	TAA
nad4	4423	5741	+	1320	ATG	TAA
Intergenic spacer 3	5742	5749	+	8	n.a.	n.a.
trnP(tgg)	5750	5816	+	67	n.a.	n.a.
Intergenic spacer 4	5817	5819	+	3	n.a.	n.a.
nad1	5820	6740	+	921	ATG	TAA
Intergenic spacer 5	6741	6761	+	21	n.a.	n.a.
trnC(gca)	6762	6817	+	56	n.a.	n.a.
Intergenic spacer 6	6818	6718	+	1	n.a.	n.a.
atp8	6819	6977	+	159	ATG	TAA
cox3	6974	7756	+	783	ATA	TAA
Intergenic spacer 7	7757	7761	+	5	n.a.	n.a.
trnS1(tct)	7762	7830	+	69	n.a.	n.a.
Intergenic spacer 8	7831	7913	+	83	n.a.	n.a.
nad5	7914	9599	+	1686	ATT	TAA
trnG(tcc)	9599	9666	+	68	n.a.	n.a.
Intergenic spacer 9	9667	9814	+	147	n.a.	n.a.
rrnL	9815	10822	+	1008	n.a.	n.a.
Intergenic spacer 10	10823	10843	+	20	n.a.	n.a.
rrnS	10844	11630	+	787	n.a.	n.a.

nad6	11626	12096	+	471	ATG	TAG
Control region	12097	12798		702	n.a.	n.a.
trnL1(tag)	12799	12863	+	65	n.a.	n.a.
Intergenic spacer 11	12864	12906	+	43	n.a.	n.a.
trnK(ttt)	12907	12973	+	67	n.a.	n.a.
Intergenic spacer 12	12974	13074	+	101	n.a.	n.a.
trnT(tgt)	13075	13136	+	62	n.a.	n.a.
Intergenic spacer 13	13137	13194	+	58	n.a.	n.a.
trnD(gtc)	13195	13257	+	63	n.a.	n.a.
Intergenic spacer 14	13258	13359	+	102	n.a.	n.a.
trnE(ttc)	13360	13425	+	66	n.a.	n.a.
trnQ(ttg)	13426	13492	+	67	n.a.	n.a.
Intergenic spacer 15	13493	13559	+	67	n.a.	n.a.
nad2	13560	14543	+	984	ATG	TAA
Intergenic spacer 16	13544	13566	+	23	n.a.	n.a.
nad3	14567	14920	+	354	ATG	TAA
Intergenic spacer 17	14921	14955	+	35	n.a.	n.a.
trnN(gtt)	14956	15022	+	67	n.a.	n.a.
Intergenic spacer 18	15023	15027	+	5	n.a.	n.a.
trnI(gat)	15028	15091	+	64	n.a.	n.a.
Intergenic spacer 19	15092	15109	+	18	n.a.	n.a.
trnS2(tga)	15110	15173	+	64	n.a.	n.a.
Intergenic spacer 20	15174	15185	+	12	n.a.	n.a.
trnM(cat)	15186	15247	+	62	n.a.	n.a.
Intergenic spacer 21	15248	15252	+	5	n.a.	n.a.
trnW(tca)	15253	15319	+	67	n.a.	n.a.
Intergenic spacer 22	15320	15331	+	11	n.a.	n.a.
trnF(gaa)	15332	15395	+	64	n.a.	n.a.
Intergenic spacer 23	15396	15420	+	25	n.a.	n.a.
trnH(gtg)	15421	15486	+	66	n.a.	n.a.
Intergenic spacer 24	15487	15487	+	1	n.a.	n.a.

trnY(gta)	15488	15551	+	64	n.a.	n.a.
trnV(tac)	15551	15613	+	63	n.a.	n.a.
Intergenic spacer 25	15614	15615	+	2	n.a.	n.a.
trnA(tgc)	15616	15681	+	66	n.a.	n.a.
Intergenic spacer 26	15682	15684	+	3	n.a.	n.a.
trnR(tcg)	15685	15737	+	53	n.a.	n.a.
Intergenic spacer 27	15738	15748	+	11	n.a.	n.a.
Complete genome	1	15748	+	15748	n.a.	n.a.

Supplementary Table 2. Gene content of the mitochondrial genome of *Trypanobia* sp.

Gene	Start position	Stop position	Strand	Size (bp)	Start codon	Stop codon
cox1	1	1536	+	1536	ATG	TAA
Intergenic spacer 1	1537	1551		15	n.a.	n.a.
cox2	1552	2241	+	690	ATG	TAA
Intergenic spacer 2	2242	2256	+	15	n.a.	n.a.
cob	2257	3393	+	1137	ATG	TAA
Intergenic spacer 3	3394	3424	+	31	n.a.	n.a.
trnL2(taa)	3425	3488	+	64	n.a.	n.a.
Intergenic spacer 4	3489	3489	+	1	n.a.	n.a.
atp6	3490	4176	+	687	ATG	TAA
Intergenic spacer 5	4177	4177	+	1	n.a.	n.a.
nad4l	4178	4474	+	297	TTG	TAA
nad4	4468	5787	+	1320	ATG	TAA
Intergenic spacer 6	5788	5792	+	5	n.a.	n.a.
trnP(tgg)	5793	5857	+	65	n.a.	n.a.
Intergenic spacer 7	5858	5873	+	16	n.a.	n.a.
nad1	5874	6794	+	922	ATG	TAG
Intergenic spacer 8	6795	6814	+	20	n.a.	n.a.
atp8	6815	6973	+	159	ATG	TAA
Intergenic spacer 9	6974	7005	+	32	n.a.	n.a.
cox3	7006	7797	+	792	ATT	TAA
Intergenic spacer 10	7798	7804	+	7	n.a.	n.a.
trnS1(tct)	7805	7871	+	67	n.a.	n.a.
Intergenic spacer 11	7872	7975	+	104	n.a.	n.a.
nad5	7976	9652	+	1677	ATG	TAA
Intergenic spacer 12	9653	9657	+	5	n.a.	n.a.
trnG(tcc)	9658	9716	+	59	n.a.	n.a.
Intergenic spacer 13	9717	9868	+	152	n.a.	n.a.

rrnL	9869	10875	+	1007	n.a.	n.a.
Intergenic spacer 14	10877	10903	+	27	n.a.	n.a.
rrnS	10904	11692	+	789	n.a.	n.a.
Intergenic spacer 15	11693	11737	+	45	n.a.	n.a.
nad3	11738	12091	+	353	ATG	TAA
Intergenic spacer 16	12092	12100	+	9	n.a.	n.a.
trnN(gtt)	12101	12161	+	61	n.a.	n.a.
Intergenic spacer 17	12162	12170	+	9	n.a.	n.a.
trnH(gtg)	12171	12232	+	62	n.a.	n.a.
Intergenic spacer 18	12233	12279	+	47	n.a.	n.a.
trnC(gca)	12280	12343	+	64	n.a.	n.a.
Intergenic spacer 19	12344	12385	+	42	n.a.	n.a.
trnD(gtc)	12386	12449	+	64	n.a.	n.a.
Intergenic spacer 20	12450	12515	+	66	n.a.	n.a.
trnA(tgc)	12516	12578	+	63	n.a.	n.a.
Intergenic spacer 21	12579	12600	+	22	n.a.	n.a.
trnY(gta)	12601	12661	+	61	n.a.	n.a.
Intergenic spacer 22	12662	12926	+	265	n.a.	n.a.
trnM(cat)	12927	12988	+	62	n.a.	n.a.
Intergenic spacer 23	12989	13254	+	62	n.a.	n.a.
trnR(tcg)	13255	13312	+	58	n.a.	n.a.
Intergenic spacer 24	13313	13495	+	183	n.a.	n.a.
trnW(tca)	13496	13559	+	64	n.a.	n.a.
Intergenic spacer 25	13560	13560	+	1	n.a.	n.a.
trnE(ttc)	13561	13627	+	67	n.a.	n.a.
Intergenic spacer 26	13628	13638	+	11	n.a.	n.a.
trnK(ttt)	13639	13701	+	63	n.a.	n.a.
Intergenic spacer 27	13702	13704	+	3	n.a.	n.a.
trnT(tgt)	13705	13767	+	63	n.a.	n.a.
trnS2(tga)	13768	13830	+	63	n.a.	n.a.
Control region	13831	14612	+	782	n.a.	n.a.

trnL1(tag)	14613	14675	+	63	n.a.	n.a.
Intergenic spacer 28	14676	14726	+	51	n.a.	n.a.
trnF(gaa)	14727	14790	+	64	n.a.	n.a.
Intergenic spacer 29	14791	14838	+	48	n.a.	n.a.
trnV(tac)	14839	14900	+	62	n.a.	n.a.
Intergenic spacer 30	14901	14922	+	22	n.a.	n.a.
trnI(gat)	14923	14985	+	63	n.a.	n.a.
Intergenic spacer 31	14986	14998	+	13	n.a.	n.a.
nad6	14999	15487	+	489	ATG	TAA
Intergenic spacer 32	15488	15525	+	38	n.a.	n.a.
nad2	15526	16542	+	1017	ATG	TAA
Intergenic spacer 33	16543	16547		5	n.a.	n.a.
trnQ(ttg)	16548	16610	+	63	n.a.	n.a.
Intergenic spacer 34	16611	16630	+	20	n.a.	n.a.
Complete genome	1	16630	+	16630	n.a.	n.a.

Supplementary Table 3: Codon usage in *Ramisyllis multicaudata* and *Trypanobia* sp. The relative synonymous codon usage (RSCU) is the number of times a codon appears in a gene divided by the number of expected occurrences under equal codon usage. If the synonymous codons of an amino acid are used with equal frequencies, their RSCU values will equal 1.

		Ramisyllis		*Trypanobia*	
**Codon**	**AA**	**ObsFreq**	**RSCU**	**ObsFreq**	**RSCU**
UAG	*	17	0,493	1	0,250
UAA	*	**52**	1,507	**7**	1,750
GCU	A	66	1,382	59	1,216
GCG	A	**5**	0,105	**6**	0,124
GCC	A	55	1,152	56	1,155
GCA	A	65	1,361	73	1,505
UGU	C	**17**	1,308	**24**	1,600
UGC	C	9	0,692	6	0,400
GAU	D	39	1,182	38	1,134
GAC	D	27	0,818	29	0,866
GAG	E	18	0,450	7	0,167
GAA	E	**62**	1,550	**77**	1,833
UUU	F	124	1,093	143	1,144
UUC	F	103	0,907	107	0,856
GGU	G	20	0,523	27	0,632
GGG	G	33	0,863	20	0,468
GGC	G	20	0,523	17	0,398
GGA	G	**80**	2,092	**107**	2,503
CAC	H	48	0,865	35	0,843
CAU	H	**63**	1,135	**48**	1,157
AUU	I	**176**	1,181	**211**	1,275

AUC	I	122	0,819	120	0,725
AAA	K	**84**	1,697	**108**	1,831
AAG	K	15	0,303	10	0,169
CUA	L	**210**	2,205	**184**	2,109
CUC	L	57	0,598	43	0,493
CUG	L	20	0,210	10	0,115
CUU	L	94	0,987	112	1,284
UUA	L	183	1,877	203	1,924
UUG	L	12	0,123	8	0,076
AUG	M	29	0,212	23	0,143
AUA	M	**244**	1,788	**299**	1,857
AAC	N	56	0,794	63	0,754
AAU	N	**85**	1,206	**104**	1,246
CCU	P	71	1,442	50	1,010
CCG	P	**5**	0,102	**9**	0,182
CCC	P	60	1,218	53	1,071
CCA	P	61	1,239	86	1,737
CAG	Q	12	0,300	1	0,034
CAA	Q	**68**	1,700	**58**	1,966
CGA	R	**31**	2,214	**42**	3,111
CGC	R	10	0,714	6	0,444
CGG	R	5	0,357	1	0,074
CGU	R	10	0,714	5	0,370
AGC	S	7	0,153	2	0,047
AGA	S	60	1,311	67	1,576
UCA	S	**105**	2,295	**143**	3,365
UCC	S	83	1,814	57	1,341
UCG	S	8	0,175	4	0,094

UCU	S	84	1,836	53	1,247
AGG	S	6		8	0,188
AGU	S	13	0,284	6	0,141
ACA	T	**113**	1,638	**123**	1,984
ACC	T	61	0,884	47	0,758
ACG	T	3	0,043	6	0,097
ACU	T	99	1,435	72	1,161
GUC	V	22	0,561	14	0,339
GUG	V	11	0,280	6	0,145
GUU	V	36	0,917	45	1,091
GUA	V	**88**	2,242	**100**	2,424
UGA	W	**68**	1,545	**87**	1,851
UGG	W	20	0,455	7	0,149
UAC	Y	65	0,855	60	0,952
UAU	Y	**87**	1,145	66	1,048

Supplementary Table 4. Matrix of gene order similarity measure obtained from CREx analysis. The higher the numbers the more similar are compared gene orders.

	L. terrestris	***P. dumerilii***	***R. multicaudata***	***Trypanobia* sp.**
Lumbricus terrestris	1326	358	2	2
Platynereis dumerilii	358	1326	2	4
Ramisyllis multicaudata	2	2	1326	134
***Trypanobia* sp.**	2	4	134	1326

Supplementary Table 5. Terminals used in the analyses, sampling sites and GenBank accession numbers. *Typosyllis* and *Syllis* species as they were originally described. WA: Western Australia; NSW: New South Wales; QLD: Queensland; Aus: Australia.

Ingroup	Sample site	18S	16S	COI
Alcyonosyllis hinterkircheri	Kimberley, WA, Australia	KM277825	KM277821	
Amblyosyllis formosa	Banyuls-sur-Mer, France	EF123834		
Amblyosyllis madeirensis	San Esteban, Asturias, Spain	JF903574		
Amblyosyllis sp. 1	Manazuru, Japan	JF913963		
Amblyosyllis sp. 2	Banyuls-sur-Mer, France	AF474284		
Anguillosyllis capensis	GenBank	GQ426601		
Anguillosyllis sp.	Costa Rica	JF903571		
Branchiosyllis cirropunctata	Green Head, WA, Australia	JF903580	JF903690	
Branchiosyllis exilis 1	Darwin, NT, Australia	JF903583		
Branchiosyllis exilis 3	Shark Bay, WA, Australia	JF903581-2		
Branchiosyllis exilis 2	Coconut Is., Hawaii, USA	JF903584		
Branchiosyllis maculata	Heron Island, QNLD, Australia	JF903585	JF903694	
Branchiosyllis sp.	La Jolla, California, USA	AF474283		
Branchiosyllis thylacine	Port Jackson, NSW, Australia	JF903586	JF913951	
Brania arminii	Cádiz, Spain	EF123831		
Brania furcelligera	Shark Bay, WA, Australia	JF903587		
Brania pusilla	Vigo, Galicia, Spain	EF123838		
Epigamia magna	Washington, USA	AF474309		
Epigamia noroi	California, USA	AF474310		
Erinaceusyllis belizensis	Cádiz, Spain	EF123823-4		
Erinaceusyllis bidentata	Shark Bay, WA, Australia	JF903589		
Erinaceusyllis cryptica	Cabo de Gata, Almería, Spain	JF913964		
Erinaceusyllis hartmannschroederae	Port Jackson, NSW, Australia	JF913965		
Erinaceusyllis horrockensis 1	Por Denison, WA, Australia	JF903590		
Erinaceusyllis horrockensis 2	Botany Bay, NSW, Australia	JF903591		
Erinaceusyllis kathrynae	Kalbarry, WA, Australia	JF903592		
Erinaceusyllis serratosetosa	Port Denison, WA, Australia	JF903593		
Eurisyllis tuberculata 1	Shark Bay, WA, Australia	JF903594		JF903787
Eurisyllis tuberculata 2	Banyuls-sur-mer, France	EF123833		EF123748
Eusyllis blomstrandi	Kaldbak, Faroe Islands	EF123887		
Eusyllis kupfferi	Kalbarry, WA, Australia	JF903595		
Exogone africana 1	Kalbarry, WA, Australia	JF903596		
Exogone africana 2	West of Angel Is., WA, Aus.	JF903597		
Exogone aristata	Port Jackson, NSW, Australia	JF903598		
Exogone dispar	Port Denison, WA, Australia	JF903599		
Exogone fustifera	Geraldton, WA, Australia	JF903600		
Exogone haswelli	Botany Bay, NSW, Australia	JF903601		
Exogone heterosetoides	Shark Bay, WA, Australia	JF903603		
Exogone heterosetosa	Sydney, NSW, Australia	JF903602		
Exogone naidina 1	Wales, United Kingdom	EF123886		
Exogone naidina 2	Wales, United Kingdom	AF474290		
Exogone naidinoides 1	Port Denison, WA, Australia	JF903604		
Exogone naidinoides 2	Sydney, NSW, Australia	JF903605		
Exogone rostrata	Port de la Selva, Girona, Spain	JF903606		
Haplosyllis sp. *1*	Shark Bay, WA, Australia	JF903607		
Haplosyllis sp. *2*	Goodes Is., QNLD, Australia	JF903608-9		
Haplosyllis sp. *3*	Coconut Is., Hawaii, USA	JF903610-KP974806		

Haplosyllis spongicola 1	Banyuls-sur-mer, France	EF123837	EF123791	EF123751
Haplosyllis spongicola 2	GenBank	AF474291		
Megasyllis corruscans	Sydney, NSW, Australia	KM277826	KM277823	KM277828
Megasyllis inflata	Port Jackson, NSW, Australia	JF913966		
Megasyllis sp. *1*	Manazuru, Japan	JF903577		
Megasyllis sp. *2*	Manazuru, Japan	JF903614		
Megasyllis mariandreworum	Botany Bay, NSW, Australia	JF903613		
Megasyllis multiannulata	Manazuru, Japan	JF903612	JF903699	
Megasyllis glandulosa	Port Denison, WA, Australia	JF903576		
Megasyllis subantennata	Kalbarry, WA, Australia	JF903578	JF903688	JF903775
Megasyllis tigrina	Jervis Bay, NSW, Australia	JF903579		
Myrianida convoluta	California, USA	AF474303		
Myrianida edwarsi	GenBank	AF474294		
Myrianida pachycera	California, USA	AF474304		
Myrianida pinnigera	Cádiz, Spain	EF123843		
Myrianida prolifera	Kristineberg, Sweden	AF474295		
Myrianida sp.	O Grove, Galicia, Spain	EF123855		
Nudisyllis pulligera	Banyuls-sur-mer, France	AF474286		
Odontosyllis australiensis	Heron Island, QNLD, Australia	JF903615-KP974807		
Odontosyllis ctenostoma	Cabo de Gata, Almería, Spain	JF903616-7		
Odontosyllis detecta	Port Jackson, NSW, Australia	JF903618		
Odontosyllis freycinetensis	Jurien Harbour, WA, Australia	JF903619		
Odontosyllis fulgurans	Port de la Selva, Girona, Spain	EF123882		
Odontosyllis gibba	Port de la Selva, Girona, Spain	EF123850		
Odontosyllis globulocirrata	Jurien Harbour, WA, Australia	JF903620		
Odontosyllis maculata	Manazuru, Japan	JF903621		
Odontosyllis polycera	Port Jackson, NSW, Australia	JF913967		
Odontosyllis pentalineata	San Diego, California, USA	JF903572		
Parahaplosyllis brevicirra	Port Jackson, NSW, Australia		JF903706	JF903784
Paraopisthosyllis alternocirra	Rottnest Is., WA, Australia	JF903623		
Paraopisthosyllis cf. *phyllocirra*	Lizard Island, QNLD, Australia	JF903624	JF903708	JF903777
Parapionosyllis elegans	San Esteban, Asturias, Spain	JF903625		
Parapionosyllis labronica	Cabo de Gata, Almería, Spain	JF903626		
Parapionosyllis sp.	Banyuls-sur-Mer, France	AF474287		
Perkinsyllis augeneri	Sydney, NSW, Australia	EF123832	EF123794	
Perkinsyllis hartmannschroederae	Port Denison, WA, Australia	JF903627		
Perkinsyllis heterochaetosa	Botany Bay, NSW, Australia	JF903628	JF913961	
Perkinsyllis koolalya	Rottnest Is., WA, Australia	JF903629	JF903709	
Pionosyllis enigmatica	Tjärno, Sweden	EF123826		
Proceraea aurantiaca	Banyuls-sur-Mer, France	AF474324		
Proceraea cornuta	GenBank	AF474312		
Proceraea misakiensis	Manazuru, Japan	JF913968		
Proceraea okadai	Washington, USA	AF474319		
Proceraea picta	Banyuls-sur-Mer, France	EF123854		
Procerastea sp.	California, USA	AF474315		
Prosphaerosyllis battiri	Rottnest Is., WA, Australia	JF903630		
Prosphaerosyllis isabellae	Port Denison, WA, Australia	JF903631		
Prosphaerosyllis longipapillata	Shark Bay, WA, Australia	JF903632		
Prosphaerosyllis magnoculata	Sydney, NSW, Australia	JF903633		
Prosphaerosyllis multipapillata	Botany Bay, NSW, Australia	JF920030		
Prosphaerosyllis xarifae	O Grove, Galicia, Spain	EF123836		
Ramisyllis multicaudata	Darwin, NA, Australia	KR604716	KR534502	KR534502
Salvatoria clavata	Port de la Selva, Girona, Spain	EF123825		
Salvatoria euritmica	Port Jackson, NSW, Australia	JF903634		
Salvatoria kerguelensis	Ningaloo, WA, Australia	JF903635		
Salvatoria koorineclavata	Rottnest Is., WA, Australia	JF903636		

Salvatoria limbata 1	Port de la Selva, Girona, Spain	EF123872		
Salvatoria limbata 2	Koster Area, Sweden	AF474289		
Salvatoria quadrioculata	Port Jackson, NSW, Australia	JF903638		
Salvatoria sp.	Cabo de Gata, Almería, Spain	JF903639		
Sphaerosyllis austriaca	Port de la Selva, Girona, Spain	EF123884		
Sphaerosyllis bardukaciculata	Sydney, NSW, Australia	EF123842		
Sphaerosyllis boeroi	Port de la Selva, Girona, Spain	EF123856		
Sphaerosyllis capensis	Ningaloo, WA, Australia	JF903640		
Sphaerosyllis densopapillata	Sydney, Australia	JF903641		
Sphaerosyllis glandulata	Port de la Selva, Girona, Spain	EF123840		
Sphaerosyllis hirsuta 1	Rottnest Is., WA, Australia	JF903642		
Sphaerosyllis hirsuta 2	Sydney, NSW, Australia	EF123870		
Sphaerosyllis hirsuta 3	Port Jackson, NSW, Australia	JF903643		
Sphaerosyllis hystrix	Port de la Selva, Girona, Spain	EF123880		
Sphaerosyllis pirifera	Port de la Selva, Girona, Spain	EF123845		
Sphaerosyllis taylori	Port de la Selva, Girona, Spain	EF123866-7		
Streptosyllis aequiseta	Pittwater, NSW, Aus	JF903644		
Streptosyllis bidentata	Port de la Selva, Girona, Spain	GU357625		
Syllides cf. *japonicus*	Port Jackson, NSW, Australia	GU357624		
Syllides convolutus	Port de la Selva, Girona, Spain	EF123829-30		
Syllides edentatus	Cabo de Gata, Almería, Spain	GU357626		
Syllides fulvus	Banyuls-sur-Mer, France	EF123839		
Syllides sp. *2*	Port de la Selva, Girona, Spain	JF903646		
Syllides sp. *3*	Ningaloo, WA, Australia	JF903645		
Syllides sp. *4*	Port Jackson, NSW, Australia	JF903647		
Syllides tam	Botany Bay, NSW, Australia	JF903648		
Syllis alternata	Port de la Selva, Girona, Spain	JF903649	JF903726	
Syllis armillaris 1	Kaldbak, Faroe Islands	AF474292		
Syllis armillaris 2	Cala Ratjada, Mallorca, Spain	JF913969-KP974809	JF903727	
Syllis cerina	Prince of Wales Is., QNLD, Aus	JF903653		
Syllis cf. *variegata*	Port Jackson, NSW, Australia	JF903655-KP974810		
Syllis compacta	Altea, Alicante, Spain	EF123846-7	EF123806	EF123772
Syllis corallicola	Port de la Selva, Girona, Spain	EF123875		
Syllis ehlersioides	Manazuru Peninsula, Japan	EF123841	EF123808	EF123773
Syllis ferrani	Port de la Selva, Girona, Spain	EF123874	EF123809	EF123775
Syllis gracilis 1	O Grove, Galicia, Spain	EF123876	EF123811	EF123778
Syllis cf. *gracilis australiensis 1*	Rat Island, Abroholos, WA, Aus	KM277831		
Syllis cf. *gracilis australiensis 2*	Cassini, Kimberley, WA, Aus	KM277830		
Syllis hyalina 1	Port de la Selva, Girona, Spain	EF123851-2	EF123818	EF123779
Syllis hyalina 2	Port Phillip Bay, Victoria, Aus.	JF903662		
Syllis krohnii	Azores Islands, Portugal	EF123859	EF155920	
Syllis marugani	Manazuru Peninsula, Japan	EF123862-3	EF123812	EF123780
Syllis nigropunctata	Port Jackson, NSW, Australia	JF903664-KP974811	JF903737	
Syllis okadai	Manazuru Peninsula, Japan	EF123857-8	EF123814	EF123783
Syllis pectinans	Newport, NSW, Australia	JF903666		
Syllis prolifera 1	Cabo de Gata, Almería, Spain	JF903667-KP974812	JF903739	
Syllis prolifera 2	Cala Ratjada, Mallorca, Spain	JF903668-KP974813		
Syllis pulvinata	Port de la Selva, Girona, Spain	JF913972		
Syllis punctulata	Maroubra, NSW, Australia	JF903669-KP974814	JF903742	
Syllis variegata	O Grove, Galicia, Spain	JF903670	EF123822	
Syllis vittata	San Esteban Asturias, Spain	JF903671		
Syllis vivipara	O Grove, Galicia, Spain	EF123848-9	EF123815	

Syllis westheidei	Port de la Selva, Girona, Spain	EF123877		EF123784
Syllis ypsiloides	Coconut Is. Hawaii, USA	JF903673		
Syllis zonata	Port Jackson, New South Wales	JF903674	JF903746	
Synmerosyllis lamelligera	Port de la Selva, Girona, Spain	EF123864		
Synmerosyllis sp.	Costa Rica, Pacific O.	JF903573		
Synmerosyllis yolandae	Port Jackson, NSW, Australia	JF913973		
Trypanobia sp.	Lizard island, QLD, Australia	KR604717	KR534503	KR534503
Trypanobia depressa	Lizard island, QLD, Australia	KR364795-6	KR364797	KR364801
Trypanosyllis coeliaca 1	Port de la Selva, Girona, Spain	EF123878	EF123816	EF123785
Trypanosyllis coeliaca 2	Sydney, NSW, Australia	JF903675	JF903749	
Trypanosyllis sp	Lizard island, QLD, Australia	KR364799-<br>KR364800	KR364797	
Trypanosyllis zebra 1	Banyuls-sur-Mer, France	JF903676	EF123817	EF123786
Trypanosyllis zebra 2	Shark Bay, WA, Australia	JF903677	JF903751	
Trypanosyllis zebra 3	Port Jackson,, NSW, Australia	JF903678-<br>KP974815	JF903752	JF903790
Typosyllis anoculata	GenBank	DQ790098		
Typosyllis bella	Abroholos, WA, Australia	JF913971		
Typosyllis antoni	Aquarium Leipzig, Germany	SRX513556	SRX513556	SRX513556
Typosyllis broomensis	Goodes Is., QNLD, Australia	JF903650-1	JF903728	
Typosyllis busseltonensis	Port Jackson, NSW, Australia	JF903652		
Typosyllis cf. *lutea*	Heron Island, QNLD, Australia	JF903654		
Typosyllis columbretensis	Port de la Selva, Girona, Spain	JF903656	EF123805	EF123771
Typosyllis crassicirrata	Port Phillip Bay, Victoria, Aus.	JF903657		
Typosyllis filidentata	Little Kianinny, NSW, Australia	KM277829		
Typosyllis garciai	O Grove, Galicia, Spain	EF123869		EF123776
Typosyllis heronislandensis	Heron Island, QNLD, Australia	JF903661	JF903734	
Typosyllis lutea 1	Sydney, NSW, Australia	EF123865		
Typosyllis lutea 2	Shark Bay, WA, Australia	JF903663	JF903736	JF903785
Typosyllis monilata	Manazuru Peninsula, Japan	EF123860-1	EF123819	EF123781
Typosyllis patriciae	Shark Bay, WA, Australia	JF903665	JF903738	JF903781
Typosyllis pigmentata	Manazuru Peninsula, Japan	EF155921		
Typosyllis setoensis	Cassini, Kimberley, WA, Aus	KM277832		
Typosyllis yallingupensis	Port Denisson, WA, Australia	JF903672	JF903744	
Virchowia clavata	Banyuls-sur-mer, France	AF474314		
Xenosyllis scabroides	Lizard Island, QNLD, Australia	JF913974-<br>KP974817	JF903753	
**Outgroup**				
Aglaophamus malmgreni	GenBank	AY996091		
Chrysopetalum debile	GenBank	EU555037		
Eulalia viridis	GenBank	AY996085		
Neanthes virens	GenBank	Z83754		
Nereimyra punctata	GenBank	DQ779661		
Sigambra sp.	GenBank	AY340444		
